# Brain–immune interaction mechanisms: Implications for cognitive dysfunction in psychiatric disorders

**DOI:** 10.1111/cpr.13295

**Published:** 2022-07-20

**Authors:** Fangyi Zhao, Bingjin Li, Wei Yang, Tongtong Ge, Ranji Cui

**Affiliations:** ^1^ Jilin Provincial Key Laboratory on Molecular and Chemical Genetic The Second Hospital of Jilin University Changchun China

## Abstract

**Objectives:**

Cognitive dysfunction has been identified as a major symptom of a series of psychiatric disorders. Multidisciplinary studies have shown that cognitive dysfunction is monitored by a two‐way interaction between the neural and immune systems. However, the specific mechanisms of cognitive dysfunction in immune response and brain immune remain unclear.

**Materials and methods:**

In this review, we summarized the relevant research to uncover our comprehension of the brain–immune interaction mechanisms underlying cognitive decline.

**Results:**

The pathophysiological mechanisms of brain‐immune interactions in psychiatric‐based cognitive dysfunction involve several specific immune molecules and their associated signaling pathways, impairments in neural and synaptic plasticity, and the potential neuro‐immunological mechanism of stress.

**Conclusions:**

Therefore, this review may provide a better theoretical basis for integrative therapeutic considerations for psychiatric disorders associated with cognitive dysfunction.

## Graphical abstract

A growing body of research suggests that cognitive dysfunction accounts for an increasing proportion of multiple psychiatric disorders. Many factors contribute to cognitive dysfunction in psychiatric disorders, with brain–immune interactions being the most common mechanism. The mechanisms of brain–immune interactions include a variety of aspects, such as the abnormal release of multiple cytokines and immune molecules, disturbed neuron–glia interactions, neuroinflammation, early‐life stress, synaptic plasticity, as well as the abnormal secretion of glial cells. Understanding the pathogenesis of cognitive dysfunction will help unravel the mystery of the pathogenesis of many psychiatric disorders.

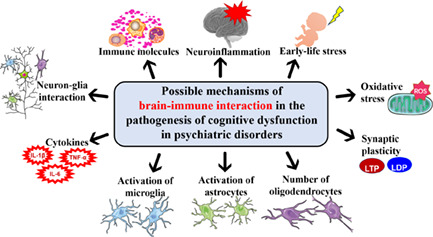

## 1 INTRODUCTION

Cognitive dysfunction is increasingly recognized as a common symptom across a range of psychiatric disorders and an essential risk element for the pathogenesis, pharmacological response and prognosis of these diseases.^1^, ^2^, ^3^ Not only that, cognitive dysfunction refers to a complex disorder involving multiple factors such as attention, memory,^4^ learning, executive functioning,^5^ psychomotor speed, computation, problem‐solving skills, comprehension as well as judgement.^6^, ^7^, ^8^ There is growing evidence that cognitive dysfunction persists even following the emotional symptomatic remission in psychiatric disorders, and that the effects are severe and long‐lasting.^9^, ^10^ Therefore, clarifying the pathophysiological mechanisms of cognitive dysfunction is of great significance to explain the mechanisms and integrative treatment of psychiatric disorders. However, the specific pathogenesis of cognitive dysfunction is still unknown. The mounting amount of research demonstrates that the two‐way communication amongst the central nervous system (CNS) and the immune system is critical for the physical proper immunological, behavioural and physiological response.^11^, ^12^ Recent advances in the multidisciplinary direction suggest that cognitive function is mediated by bidirectional interactions between the brain and immune system.^12^, ^13^, ^14^, ^15^ A growing number of researchers believe that brain–immune interactions play an integral role in the pathogenesis and treatment of psychiatric disorders.^16^, ^17^ It is becoming increasingly clear that pathophysiological mechanisms of brain–immune interaction in cognitive dysfunction involve several specific immune molecules and their associated signalling pathways, impairments in neural and synaptic plasticity (neuron–glia integrity), as well as the potential neuro‐immunological mechanism of stress.^18^, ^19^, ^20^, ^21^ Under conditions of normalcy, immune mechanisms are triggered by environmental or psychological stimuli that regulate communication pathways from the peripheral immune system to the brain, as well as signals generated by immune‐like processes involving neuron–glia communication.^22^ Studies have demonstrated that various psychiatric disorders resulting from cognitive dysfunction can stimulate brain immune cells, microglia, and astrocytes to trigger signalling pathways through the activation of innate immune receptors. Toll‐like receptors (TLRs) and NOD‐like receptors (NLRs), ultimately produce pro‐inflammatory cytokines and chemokines, leading to neuroinflammation.^23^, ^24^ This neuroinflammation may lead to cognitive dysfunction and increased susceptibility to psychiatric disorders. Research in the late 1980s clearly showed that cells can produce nitric oxide (NO) a gas molecule that has a range of functions in the CNS, such as regulating synaptic plasticity and is also involved in cardiovascular, immune, and neurological regulation, rather than just being a toxic pollutant.^25^ NO, CO, and hydrogen sulphide gases are gaseous signalling molecules, and many studies now suggest that certain physiological levels of these gases have neuroprotective effects.^25^ In the CNS, NO and CO perform many functions, such as regulating synaptic plasticity, sleep–wake cycles, and hormone secretion, and more interestingly, nitric oxide plays an important role in the mechanisms of cell death or survival.^25^ It is reported that cellular stress is related to various psychiatric disorders. Under stressful conditions, intracellular homeostasis processes may be disrupted, which may induce a process of unfolded protein response (UPR) in the subcellular lumen of the endoplasmic reticulum (ER)^26^ Some experimental studies have shown that ER stress pathway is involved in cognitive decline and cognitive dysfunction.^27^ In this review, we aim to summarize the mechanisms of brain–immune interactions underlying cognitive dysfunction in psychiatric disorders from the perspective of individual, cellular and molecular levels to improve our comprehension of the brain–immune interaction in cognitive dysfunction and offer new insights and potential treatments for a variety of psychiatric disorders.

## 2 THE RELATIONSHIP BETWEEN COGNITIVE DYSFUNCTION, BRAIN IMMUNE, AND PSYCHIATRIC DISORDERS

In the broad sense of immunology, ‘immune’ refers to a series of behavioural changes that occur in a living organism that recognizes ‘self’, eliminates ‘nonself’ and keeps itself stable.^28^ The immune system is distributed throughout the entire body, destroying and rejecting antigens (e.g. pathogens and their products, tumour cells) or heterogeneous substances (e.g. ageing self‐cells, damaged cells, etc.) that enter the body to maintain the balance and stability of the internal homeostasis.^28^ Currently, the CNS (brain) is regarded as an immune‐privileged site because it can tolerate the introduction of antigens without causing an inflammatory immune response. In the past, peripheral immune cells of the brain were only reported in pathological conditions, such as acute brain inflammation or acute infections; however, current studies have found that bone marrow‐derived immune cells are identified to have an instrumental role in neuroprotection, repair of brain injury, and healthy brain synaptic plasticity.^29^ It is becoming increasingly clear that there is an intricate connection between the brain and the immune system. The psychiatric disorder of depression in the brain, for example, is closely linked to two‐way communication between the immune system and the brain.^30^, ^31^ Experiments by Kipnis et al. showed that the cognitive abilities of mice were impaired in the absence of mature T cells, but this effect could be restored by passive T‐cell transfer, suggesting a link between brain maintenance and peripheral adaptive immunity and that the ability to cope with neurodegenerative psychiatric disorders may depend on CD4+ T cells.^32^ There is growing evidence that healthy CNS cognition, plasticity, neurogenesis and coping with psychiatric stress are dependent on the immune system and that the mechanisms of immune effects in the brain are diverse that involve a wide variety of cells and signalling pathways. In addition to lymphocytes, microglia, and intrinsic myeloid cells of the brain, neuronal cells (neurons, oligodendrocytes, and astrocytes) also play key immune functions in the brain.^33^, ^34^ Cytokines are one of the most important signalling molecules in the immune system. Cytokines, when recognized by neuronal cells, may influence higher brain functions, such as cognitive dysfunction and memory, and in turn, neurotransmitters and neuropeptides, mediators of the brain nervous system, may influence immune cells.^29^, ^35^ The expression of pattern recognition receptors (PRRs) receptors (TLRs and NLRs) and cytokine receptors on neurons has been shown to provide molecular substrates for pathogen‐associated molecular patterns (PAMPs) that regulate both immune and neuronal functions.^36^, ^37^ Inflammation is a protective response of the organism to invasion or other injuries by pathogens and is also a common response of the immune system. Although transient acute inflammation is beneficial to the organism, a sustained inflammatory response is tightly associated with tissue dysfunction and the pathology of many psychiatric disorders. Numerous studies have shown that repeated physical exercises not only improve immune surveillance and immunity but also allow immune cells in the CNS to acquire an anti‐inflammatory phenotype and that the anti‐inflammatory effects induced by physical activity are significant in improving cognitive function and dementia.^38^, ^39^ Thus, the role of the triad of cognitive dysfunction, brain immune, and psychiatric disorders merits further discussion.

## 3 COGNITIVE DYSFUNCTION AND IMMUNE MOLECULAR AS WELL AS RELEVANT SIGNALLING PATHWAY

In recent years, the views on the pathogenesis of cognitive dysfunction have been changing, and now it is believed that the immune molecular and relevant signalling circuits perform a pivotal function in the etiopathogenesis of cognitive dysfunction.^40^, ^41^ Findings in animals and patients with psychiatric disorders suggest that neuroinflammation activates immune molecular and relevant signalling pathways, which contribute to behavioural symptoms and changes in psychiatric disorders.^42^ Several experiments have shown that the inflammatory response of the CNS (brain) can not only cause or increase tissue damage but also promote neuroprotection and repair. This dual effect, the counterbalance between the damaging and protective aspects, ultimately dictates the consequences of immune interactions in the brain.^43^, ^44^, ^45^ Two‐way communication between the immune system and the brain occurs in different organs, involving a wide range of cells and mediators.^46^, ^47^ Brain–immune interaction between immune molecular and cognitive impairment may contribute to our understanding of the pathophysiology of psychiatric disorders. It has now been proved that many of the neuromodulators and immunoreactive substances (immune molecules, complement, interferon, interleukin, tumour necrosis factor, etc.) are produced by the immune system.^46^, ^48^ Insight into the effects of immune molecules and their associated pathways on cognitive dysfunction may provide promising therapeutic targets for the treatment and prevention of psychiatric disorders.

### 3.1 Cognitive dysfunction, *oxidative stress, vitagenes*, inflammatory cytokines, and psychiatric disorders

As mediators of our immune system, cytokines are abundant in the brain, transmitting messages to the brain, coordinating appropriate physiological and behavioural responses and protecting neurons from potentially toxic circulating substances.^49^ Cytokines also regulate the initiation, proliferation and suppression of inflammatory responses by regulating the activation, migration and proliferation of immune cells and by generating other damage‐inducing molecules.^50^ Several cytokines are approved for the treatment of diverse neuropsychiatric disorders, improving emotion and cognitive symptoms and other behavioural abnormalities in patients.^13^ Recently, extensive investigations have demonstrated that cytokines are involved in various pathophysiological processes as key inflammatory mediators linking the brain and the immune system, but the exact mechanisms remain unclear.^51^, ^52^ There is accumulating evidence that neuroinflammation is a vector of the neuroendocrine, neurotransmission, neurogenesis, as well as stress responses, and these interactions are thought to be risk factors for the onset of cognitive dysfunction. Inflammation activates adaptive immune responses primarily by stimulating antigen‐specific T and B lymphocytes and their regulatory immune transmitters (pro/anti‐inflammatory cytokines).^53^ Neuroinflammation refers to the inflammatory response within the brain or spinal cord, mediated primarily by cytokines, chemokines, as well as reactive oxygen species (ROS).^54^ Cytokines induce pro‐inflammatory cytokines that interact with the CNS and brain in a cytokine network, to influence almost all aspects of behaviour, from neurotransmitter metabolism, neuroendocrine function and synaptic plasticity, to emotion regulation, motor activity and motivational apprehension.^55^

**FIGURE 1:**
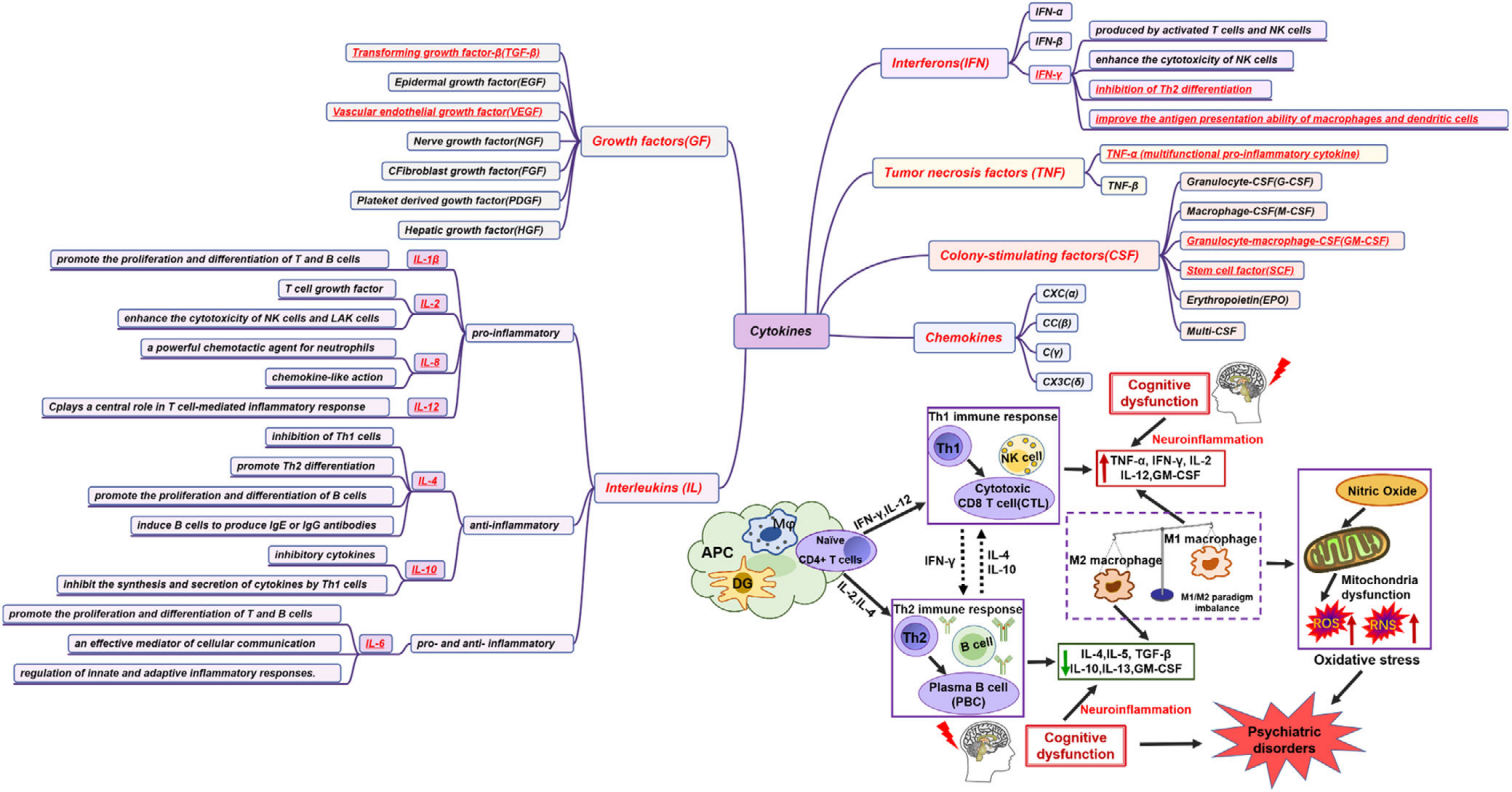
Detailed classification of cytokines and possible mechanisms by which inflammatory cytokines may play a role in cognitive dysfunction (based on the balance between M1 and M2 phenotypes of macrophages). Naïve CD4+ helper T (Th) cells (CD4+ Th0 cells) are presented by the antigen‐presenting cells (APCs) such as macrophages, dendritic cells, and B cells into CD4 helper T (Th) cells. CD4 helper T (Th) cells further differentiate into subsets of Th1 or Th2 effector cell subsets, which produce different types of (pro/anti‐inflammatory) cytokines and regulate contrasting immune responses. The production of reactive oxygen species (ROS) and reactive nitrogen species (RNS) and the uncoupling of nitric oxide synthase (NOS) may be the two main mechanisms of oxidative stress, and this mechanism is closely associated with psychiatric disorders caused by cognitive dysfunction. APC, antigen‐presenting cells; DC, dendritic cells; Mφ, macrophages; NO, nitric oxide synthase; RNS, reactive nitrogen species; ROS, reactive oxygen species

Cytokines are classified in many ways and are generally classified into six categories based on their structure and biological function: interleukins (IL), tumour necrosis factor (TNF), interferons (IFN), cytokines, growth factors (GF) and colony‐stimulating factors (CSF).^56^, ^57^, ^58^ The detailed classification and function of each cytokine are shown in Figure 1. CD4+ T‐helper (Th) cells can also be divided into different Th subsets based on the type of cytokines they produce.^59^ Cytokines also can be classified into Th‐1 and Th‐2 according to the source of lymphocyte T‐helper production.^60^ Cytokines released by Th‐1 lymphocytes and their inhibitors activate macrophages, NK cells, neutrophils and cytotoxic lymphocytes, enhancing cell‐mediated immune responses, whereas Th‐2 lymphocytes boost humoral responses by activating cells to express antibodies.^60^ Macrophages are the most common mononuclear phagocytes that perform a vital function in the immune system, exerting a critical impact on inflammatory conditions.^61^ The two types of macrophages were initially named classically activated macrophages (M1) and alternatively activated macrophages (M2).^62^, ^63^ From the macrophage domain, there are two activation states, which is the M1 state as a proinflammatory state, and alternate activation, which is the M2 state associated with the repair. Typically, ‘classically activated’ myeloid cells (LPS ± IFN‐γ) have been referred to as ‘M1’, whilst ‘alternatively activated’ cells are referred to as ‘M2’.^64^, ^65^ M1 macrophages boost pro‐inflammatory cytokine synthesis and reduce anti‐inflammatory cytokine production via activating the nuclear factor kappa‐light‐chain‐enhancer of activated B cells (NF‐κB); however, the alternative M2 activation causes activation of IκB, resulting in the inactivation of NF‐κB and subsequent inhibition of pro‐inflammatory genes, as well as increased expression of TGF‐β and other anti‐inflammatory molecules.^66^, ^67^ M1 macrophages, also known as classical macrophages, are macrophages that can produce pro‐inflammatory cytokines and have strong characteristics of microbicidal properties, but this specificity is also easy to cause tissue destruction.^68^ Activation of M1 microglia also leads to the upregulation of CD16/32 and CD68, which adversely affects neurons and further exacerbates tissue inflammation and damage.^67^ M2 macrophages, also known as alternatively activated macrophages, are stimulated by several factors such as CSF‐1, IL‐4, IL‐10, IL‐13, TGF‐β, VEGF, EGF, arginase‐1 (Arg‐1), CD206, and fungi and helminth infections and other factors, which can promote the M2 subpopulation polarization.^62^, ^69^ M2 macrophages promote inflammatory remissions through anti‐inflammatory factors that inactivate pro‐inflammatory cell phenotypes and re‐establish homeostasis, which lead to neuroprotection and promote recovery and remodelling. Numerous studies have shown that the M2 phenotype facilitates restorative actions, including neurogenesis, remodelling of the axon, angiogenesis, oligodendrogenesis, as well as myelin regeneration.^70^ Later, macrophages were divided into pro‐and anti‐inflammatory phenotypes according to the differences in functional modification of macrophages by T‐helper 1 (Th1) cytokines and Th2 cytokines.^71^, ^72^ Th1 cells can cause macrophage activation, inflammation, and tissue damage, whilst Th2 cells mediate humoral immune response and inhibit a variety of inflammatory functions of macrophages. Generally, Th‐1 cells mainly produce pro‐inflammatory cytokines, whereas Th‐2 cells primarily produce anti‐inflammatory cytokines.^53^ Pro‐inflammatory cytokines are secreted by activated immune cells (monocytes and macrophages) by activating additional cellular constituents in the inflammatory response, whereas anti‐inflammatory cytokines help to reduce the inflammatory immune reaction.^53^

Th‐1 lymphocytes are characterized by intense phagocytic activity and secreting IL‐1β, IFN‐γ, TNF‐α, IL‐2, IL‐6 as well as IL‐12, Th‐2 lymphocytes are characterized by secreting IL‐4, IL‐9, IL‐10, IL‐13 as well as TGF‐β and stimulating type 2 immunity.^73^ CD4+ T‐helper (Th) cell activation in the CNS serves an instrumental function in the regulation of immune responses, inflammation, and eventually the remedy of various psychiatric disorders.^74^ Clinical studies have shown that inflammatory markers (IL‐1β, TNF‐α, and IL‐6) are significantly elevated in the blood and cerebrospinal fluid of patients with psychiatric disorders such as MDD.^75^ The inflammatory response induced by elevated inflammatory markers can be involved in synapse formation and mapping, as well as long‐term potentiation (LTP) and neurogenesis.^76^, ^77^ The balance between M1/M2 activation is important for maintaining health and resistance to disease. It has been suggested that the overactivation of M1 and inactivation of M2 are important in the aetiology and pathogenesis of psychiatric disorders. The immune system is generally regulated through suppression of microglia M1 activation and promotion of microglia M2 activation. M1 phenotype impairment and M2 phenotype recovery provide a prospective therapy for modulating microglia/macrophage polarization to treat cognitive dysfunction in psychiatric disorders.^78^ Clarifying the mechanisms that facilitate the transition from M1 to M2 in cognitive dysfunction possibly offers novel approaches to treatment for better outcomes.^78^

The early mediators in the inflammatory process are triggered by IL‐1, IL‐6 as well as TNF‐α, whilst effector mediators are produced by proteases, ROS, arachidonic acid metabolites, nitric oxide (NO) and carbon monoxide (CO), etc. Three NO synthase isomers of the NOS family (iNOS, eNOS and nNOS) are responsible for the synthesis of NO, and these enzymes catalyse the conversion of l‐arginine to l‐citrulline plus NO in the presence of oxygen.^25^ Levels of inducible NO (iNOS) were elevated upon exposure to IFN‐γ, TNF‐α and cellular or bacterial debris.^79^, ^80^ NO is a short‐lived lipophilic gas molecule that is critical in various physiological and developmental processes, its ability to cross all barriers of biological membranes without being able to react with different intracellular/extracellular targets, forming a series of reactive nitrogen species (RNS).^81^ The RNS includes free radicals, such as NO and nitric dioxide (NO2), as well as non‐free radicals, such as nitrous acid (HNO2) and dinitrogen tetroxide (N2O4).^82^ Physiological amounts of NO are neuroprotective, whilst higher concentrations are neurotoxic. If the cell is in a pro‐oxidant state, NO can undergo redox reactions to form toxic compounds that ultimately lead to cell damage.^25^ Abnormal vascular NO production and transport lead to endothelial dysfunction, which ultimately leads to various psychiatric disorders. A wide range of disorders of the nervous and immune systems have been associated with the excessive production of NO.^83^ Numerous studies have shown that NO and RNS are involved in the pathogenesis of neurodegenerative diseases. The physiological effect of the imbalance between the production and elimination of reactive oxygen species (ROS) on cells is called ‘oxidative stress’. More importantly, various ROS‐mediated effects protect cells from ROS‐induced oxidative stress and re‐establish or maintain ‘redox homeostasis’.^84^ Mitochondria are thought to be a correlated origin of ROS and are exposed to RNS, and there is growing evidence that mitochondrial dysfunction and ROS/RNS levels are interrelated, though in a cell‐ and environment‐dependent manner.^85^ The salutogenic effects of ROS/RNS occur at a low/moderate concentration and are associated with physiological roles in the cellular response to nitrogen‐oxygen, such as defence against infectious agents and in the function of various cellular signalling pathways. The redox state of the body is maintained through a balance between a series of antioxidant systems and the production of ROS/RNS, which are used to regulate a variety of CNS function.^84^ Poorly studied redox systems of enzymes are associated with the plasma membrane and may be involved in regulating oxidative stress levels.^86^ The expression and activity of enzymes in the plasma membrane redox system (PMRS) alter the response to physiological challenges. PMRS is well regulated in the hormetic response of neurons and other cells to a range of stimuli that increase oxidative stress.^87^ Oxidative stress leads to the accumulation of damaged macromolecules and a series of physiological changes in age‐related neurodegenerative diseases, whereas the PMRS appears to attenuate oxidative stress as a compensatory mechanism during ageing.^86^ Mitochondrial dysfunction may contribute to the development of neurodegenerative diseases. During mitochondrial dysfunction, the PMRS appears to act as a protective effect, providing a survival mechanism for cells by reducing oxidative stress.^86^ The upregulation of PMRS activity contributes to cell survival and membrane homeostasis under stress conditions.^86^ Further research on PMRS might offer not only information about neurodegenerative diseases in the elderly but also therapeutic targets for the prevention and treatment of age‐related neurodegenerative diseases.

The discoveries in cellular stress signalling have provided a new understanding of the various processes that regulate the cellular stress response, and it is believed that the brain detects and defeats oxidative stress through a complicated network of ‘longevity assurance processes’ that are linked to the expression of genes known as vitagenes.^88^, ^89^ The cellular stress response entails the activated pro‐survival pathways and the generation of molecules (heat shock proteins, glutathione, bilirubin) with anti‐oxidant and anti‐apoptotic activities, which are under the control of protective genes—vitagenes.^90^, ^91^ The cellular resistance to stress and the strongly conserved cytoprotective mechanisms are also known as the heat shock response. Heat shock proteins (HSP) play a pivotal role in the folding and repair of damaged proteins, and they promote cell survival and prevent apoptosis. HSP is delivered between cell types in the nervous system, and the provision of exogenous HSP at the site of neural injury may be an effective tactic for maintaining neuronal viability, a strategy that has been validated in many model systems.^90^, ^92^ Heat shock proteins serve as important members of the vitagene network in neuroprotection and redox proteomics as a useful tool to study redox‐regulated stress‐responsive vitagenes.^89^ The vitagene system, one of the major intracellular redox systems involved in neuroprotection, is emerging as a potential target for novel cytoprotective interventions.^93^ Studies have shown that vitagenes can encode the Hsp32, Hsp70, thioredoxin and sirtuin protein systems and heme oxygenase‐1.^91^, ^94^ Endogenous cellular defence pathways (sirtuin and Nrf2 [Nuclear factor‐erythroid 2 p45‐related factor 2] and related pathways) mediate hormetic dose responses and integrate adaptive stress responses in the prevention of neurodegenerative diseases.^87^ Heme oxygenase, located within the endoplasmic reticulum (ER), is a dynamic sensor of cellular oxidative stress and regulator of redox homeostasis in the entire phylogenetic spectrum, acting in association with NADPH cytochrome P450 reductase to oxidize heme to bilirubin, CO and free ferrous iron.^87^ CO is a gaseous second messenger that is produced in biological systems when heme oxygenase is oxidative catabolism. The promotion of ROS‐mediated signalling by CO may depend on CO concentration and exposure time, the localization of heme proteins, or the specificity of the redox response.^87^ Even though high concentrations of CO are toxic, ROS produced in response to exogenous low doses of CO may affect cellular respiration and eventually lead to adaptation.^95^ NO is produced in small quantities to regulate local brain metabolism, neurotransmitter release, gene expression and exerts a crucial role in synaptic plasticity and morphogenesis, but in the case of excessive formation, NO is an essential mediator of neurotoxicity in various diseases of the nervous system.^87^, ^96^ CO and NO are essential in the regulation of CNS functions, and impaired CO and NO metabolism lead to abnormal brain functions. Hormesis is the most effective endogenous protective mechanism against antioxidant damage, and it is a dose–response phenomenon featuring low‐dose stimulation and high‐dose inhibition, which can be graphically indicated by an inverted U‐shaped dose–response/J‐shaped or U‐shaped dose–response.^90^ Hormesis can be induced either by direct stimulation or by overcompensation for disruptions in homeostasis, and the induction of adaptive responses by previous exposure to a variety of low‐level stressors has been widely investigated and has been proven to be reliable in protecting the nervous system from a variety of neurodegenerative diseases.^97^, ^98^ Hormetic dose–response has been widely studied in neuroscience research, such as neurodegenerative diseases,^90^ in addition to various antidepressants,^99^ anxiolytic drugs,^100^ and memory‐enhancing drugs. The hormetic dose–response provides a new interpretation of the dose–response, where the typical endpoints measured at high doses in a toxicological setting show cellular damage; however, when the dose is reduced below the threshold, the low‐dose stimulation is more probably a manifestation of an adaptive response that is consistent with a measure of biological performance as may be seen in the cases of modest increases in longevity, cognition and other biomedical endpoints of interest.^87^, ^101^ The consistency of the hormetic dose–response of various biological models suggests that this dose–response may be a manifestation of plasticity in biological systems and that essentially all biological models have the same quantitative characteristics as the dose–response in response to imposed stress.^87^ The hormetic dose–response provides a quantitative indicator of biological plasticity at multiple levels of biological organization, making it a core biological element for improving biological cell survival.^87^ Indeed, the preconditioning signal leading to cellular protection through hormesis is an important redox‐dependent ageing‐associated to free radicals species accumulation,^25^ The free radical species NO not only regulates neuronal proliferation, survival and differentiation, but it is also involved in synaptic activity, neural plasticity as well as memory function, and it also regulates cell differentiation and survival in the brain by modulating transcription factors and genes.^102^ NO activates important survival pathways involved in Akt (protein kinase B) and CREB (cyclic AMP‐responsive‐element‐binding protein).^25^S‐nitrosylation of NMDA (N‐methyl‐d‐aspartate) receptor subunit or the active site of caspases is responsible for the neuroprotective effect of physiological amounts of NO.^103^ NO also induces heme oxygenase 1, a crucial enzyme in the cellular stress response.^104^ NO becomes detrimental when cells are under oxidative stress or when there is excessive production of NO, which may undergo redox reactions and form toxic RNS that ultimately lead to cellular damage. The intracellular redox state may be a key factor in determining whether NO is toxic or protective in brain cells. The mechanism of action of NO as a pro‐ or anti‐inflammatory agent depends on the complexity of the chemical nature of NO in biological systems.^87^, ^105^ For example, natural antioxidants, such as polyphenols, have been proposed for the prevention or treatment of the neurodegenerative disease—Alzheimer's disease because of their ability to counteract NO‐induced damage in vitro.^106^

The vitagene system, an important intracellular redox system, is emerging as a potential neurostimulatory target for new cytoprotective interventions. Besides, vitagenes play a crucial role in the cellular pathway of protection against oxidative stress.^91^ Oxidative stress is defined as elevated intracellular levels of ROS, leading to lipid, protein, and DNA damage; however, elevated ROS also acts as signalling molecules to maintain physiological functions, a process known as redox biology.^107^ In general, all redox biology is interconnected and regulated in various ways, including mainly the antioxidant networks and vitagene networks. A variety of nutrients, such as taurine, vitamin E, selenium, carotenoids, etc., have been shown to affect antioxidant defence and vitagene networks, helping to maintain redox balance.^108^ The regulation of endogenous cellular defence mechanisms through the vitagene system may represent an innovative approach to the treatment of psychiatric disorders.^90^ During periods of oxidative stress, the vitagene network operates as a defence system in the brain, which opens new perspectives for the treatment of brain ageing and neurodegenerative disorders.^25^, ^87^, ^109^

There is growing evidence for the concept of oxidative stress‐driven neuroinflammation as an early pathological feature of neurodegenerative diseases.^110^ Neuroinflammation, a complicated host response to tissue damage or infection, is believed to be a key mechanism leading to CNS damage and has been identified to be an appealing therapeutic target for the prevention of cognitive impairment in psychiatric disorders.^111^ In other words, neuroinflammation is typical of all neuropsychiatric disorders and is a response to the CNS homeostasis involved in normal brain development and neuropathological processes, and therefore inflammation can be either beneficial or harmful. The imbalance that exists between pro‐and anti‐inflammatory activity is the most prominent feature in defending and restoring the integrity of the injured CNS and protecting neurons from damage. Under normal physiological conditions, cytokines supply nutritional support for neurons and reinforce neuronal integrity, but under stressful conditions (oxidative stress), overproduction of cytokines can lead in part to depression‐like behaviours and cognitive dysfunction in vivo.^55^, ^77^ There is increasing evidence that the imbalance between pro‐and anti‐inflammation is involved in the pathogenesis of many human diseases so the two balance also plays a major role in brain–immune interactions in humans. Inflammatory cytokines are generally considered to be markers of activation of brain–immune interactions.^112^ It is suggested that the cytokine‐mediated activation of the inflammatory response system might take an active part in the pathogenesis of cognitive dysfunction.

As a component of the cell membrane of Gram‐negative bacteria, lipopolysaccharide (LPS) has been used in many studies to mimic infection because it triggers a rapid and well‐characterized immune response and induces depressive behaviour in animal models.^113^ The current research suggests that the bacterial endotoxin LPS has multiple effects on cognition and neuronal integrity and that multiple inflammatory cytokines would be responsible for these changes.^114^ Experimental studies from rodents have shown that inflammatory mediators and pro‐inflammatory cytokines are upregulated in peripheral tissues and the CNS.^115^, ^116^ Preliminary studies suggest that inhibition of pro‐inflammatory cytokines or their signalling circuits ameliorates depressive symptoms and enhances response to traditional antidepressant treatments.^75^, ^117^, ^118^ The hippocampus is one of the brain regions closely associated with psychiatric disorders. Numerous studies have concluded that the hippocampus has many cytokine receptors, which makes it vulnerable to high concentrations of pro‐inflammatory cytokines during neuroinflammation.^119^ Pro‐inflammatory cytokines have a detrimental effect on the regulation of hippocampal neurotransmitter signalling, ultimately leading to excitatory neuronal damage and cognitive dysfunction.^118^ Recent studies have used the mice behavioural tests, western blot and immunofluorescence experiments, respectively, to detect changes in cognitive behaviour, protein expression and pro‐inflammatory cytokines associated with cognitive function in the mice hippocampus, as well as activation of microglia in the hippocampal dentate gyrus region.^120^, ^121^ The results suggest that cognitive dysfunction is caused by overproduction of proinflammatory cytokines and hyperactivation of microglia. Anti‐inflammatory cytokines regulate the duration and intensity of behavioural symptoms of the disease, possibly via inhibition of pro‐inflammatory cytokine production and attenuation of their signalling.^122^, ^123^ Anti‐inflammatory therapy would be an important treatment for cognitive dysfunction. Future treatment of cognitive impairment in psychiatric disorders may be aimed at the immune system through the action of inflammatory cytokines.

### 3.2 Cognitive dysfunction, immune molecular signalling pathway as well as psychiatric disorders

The complex interactions between the brain and the immune system may influence the onset of psychiatric disorders. A growing body of research suggests that immune responses (innate and adaptive immunity) play an integral role in various psychiatric disorders. Uncontrolled immune responses can exacerbate the immunopathology of psychiatric disorders. Immune molecules are usually divided into membrane surface immune molecules and humoral immune molecules. The immune molecules on the membrane surface include cell surface membrane receptors (pattern recognition receptor[PRR]), major histocompatibility complex (MHC) antigen, human leukocyte antigen (HLA), and cell adhesion molecules (CAM). Humoral immune molecules usually refer to antibodies, complement (C1, C3), and cytokines (IL‐1, IL‐6, chemokine, TGF‐β, TNF, IFN). A large body of research data has shown that immune molecules (especially inflammatory cytokines) and their associated signalling pathway are a complex and important set of ways that influence the brain and behaviour. The equilibrium amongst pro‐and anti‐inflammatory cytokines, as well as their associated signalling pathways, is a prospective therapeutic target for cognitive impairment.

Cognitive dysfunction caused by multiple psychiatric disorders may activate an inflammatory response that is initially mediated by innate pattern recognition receptors (PRRs), which initiate specific immune responses by recognizing specific pathogen‐associated molecular patterns (PAMP). Recognition of PRRs is a common feature of the innate immune system. The family of PRRs includes the nucleic acid‐specific TLRs, nucleotide‐binding oligomerization domain NLRs, retinoic acid‐inducible gene I (RIG‐I)‐like receptors (RLRs), and C‐type lectin receptors (CLRs), and the intracellular signalling cascades triggered by these PRRs may be associated with a variety of psychiatric disorders.^124^ TLR signalling is a prominent inflammatory pathway, and TLR expression and TLR signalling regulators are associated with MDD. The TLR4/NF‐κB signalling pathway plays an important role in neuroinflammation‐induced cognitive dysfunction, and Zhang et al. showed that cognitive impairment in LPS‐induced neuroinflammatory mice was associated with TLR4.^125^ The NLRs have an important role in the regulation of cognition, anxiety and hypothalamic–pituitary–adrenal (HPA) axis activation.^126^ The complement cascade is an imperative component of the innate and adaptive immune system. Complement is a critical intermediate link between innate and acquired immune defences. Complement activation occurs mainly through three different pathways (classical pathway, alternative pathway, and lectin pathway), each leading to a common terminal pathway.^127^ Complement activation leads to the release of a variety of biologically active molecules that contribute to immune surveillance and tissue homeostasis. Complement activation is also responsible for the pathogenesis of various psychiatric disorders, and studies have found that the brain is also susceptible to complement‐mediated damage. The complement cascade has an essential function in microglia‐mediated synaptic refinement during brain development.^128^ Synaptic loss in Alzheimer's disease (AD) is associated with cognitive decline, and studies suggest that complement C3, which is elevated in AD, colocalizes with neuritis plaques and may contribute to the clearance of Aβ by microglia in the brain.^128^, ^129^

**FIGURE 2:**
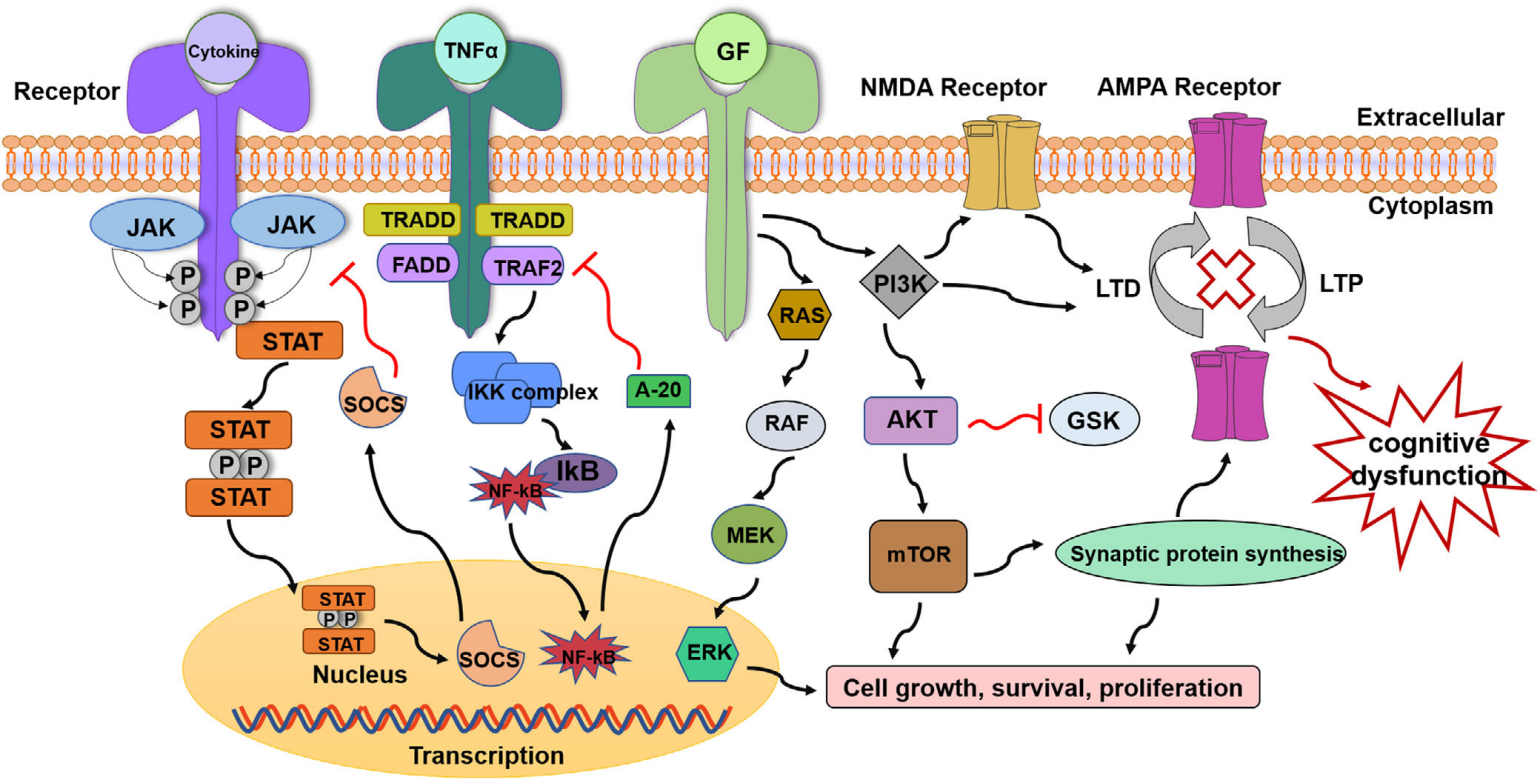
A diagram of the three most common cytokine pathways concerning cognitive dysfunction in psychiatric disorders. The PI3K/Akt/mTOR signalling pathway and the Ras/Raf/MEK/ERK signalling pathway have been identified as promising therapeutic targets for psychiatric disorder therapy. Amongst them, the mTOR can promote the synthesis of synaptic proteins, and PI3K stimulation can enhance NMDA‐dependent LTD and stimulate the exocytosis of AMPA receptors, contributing to the enhancement of excitatory synaptic transmission. AMPA, alpha‐amino‐3‐hydroxy‐5‐methyl‐4‐isoxazole‐propionic acid; Akt, protein kinase B; ERK, extracellular signal‐regulated kinase; FADD, Fas‐associated death domain protein; GF, growth factor; GSK, glycogen synthase kinase; IKK, IκB kinase; JAK, Janus kinase; LTD, long‐term depression; LTP, long‐term potentiation; mTOR, mechanistic target of rapamycin; NMDA, N‐methyl‐d‐aspartate; NF‐κB, nuclear factor kappa‐light‐chain‐enhancer of activated B cells; PI3K, phosphatidylinositol‐4,5‐bisphosphate 3‐kinase; SOCs, suppressor of cytokines; STAT, signal transducers and activators of transcription; TARDD, TNF‐receptor 1‐associated death domain protein; TRAF2, TNF receptor‐associated factor 2

**FIGURE 3:**
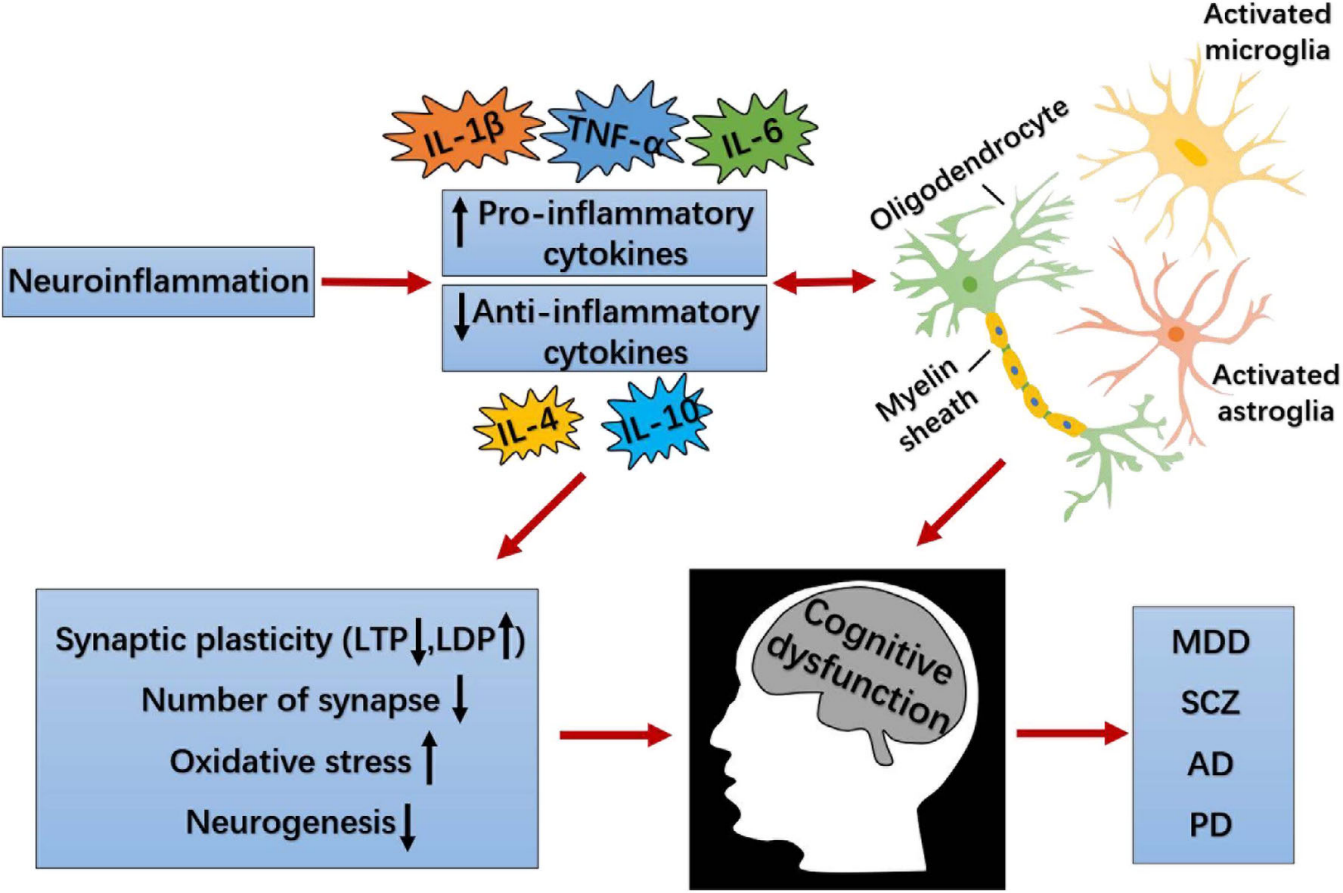
Schematic diagram of the relationship between cognitive dysfunction and psychiatric disorders. AD, Alzheimer's Disease; MDD, major depressive disorder; PD, Parkinson's Disease; SCZ, schizophrenia

Cytokines are critical modulators of immunity. Cytokines act as intercellular transmitters to regulate the immune system as well as the inflammatory response. Generally, four types of cytokine receptors are available: (1) those that activate the Janus kinase and signal transducer and activator of transcription (JAK–STAT) pathways for most cytokine families (Figure 2); (2) receptors for activating NF‐κB and mitogen‐activated protein (MAP) kinases for the TNF‐α family and IL‐1 family (Figure 2); (3) the cytoplasmic domain contains the growth factor (GF) receptor (Figure 2) of the tyrosine kinase domain (typically signals via the Ras and the extracellular signal‐regulated kinase [ERK] pathway)^130^; and (4) transforming growth factor (TGF)‐β receptors. Studies suggest that over 50 cytokines signal through the JAK/STAT pathway to induce inflammation and regulate immune responses.^131^ The JAK–STAT pathway, also known as the IL‐6 signalling circuit, is a signal transduction circuit stimulated by cytokines.^132^ Most cytokines promote gene transcriptional regulation through the JAK–STAT pathway, whilst their signalling is generally suppressed by the suppressors of the cytokine signalling (SOCS) family of proteins.^133^ The STAT family in the cytoplasm is a downstream target of JAKs, which are amongst the transcription factors essential for cytokine activation in the immune response.^132^ The SOCS proteins modify the activation of downstream genes by regulating the output of activated STATs and modifying extra diversity into the JAK–STAT pathway.^133^ Recently, M1 macrophages were proven to upregulate the expression of an intracellular protein called SOCS3 and initiate inducible nitric oxide synthase (NOS2) or iNOS to produce NO.^68^ There is evidence that M2 polarization downregulates the activity of NF‐κB and STAT1, thereby acting as a common mechanism to limit inflammatory development(Figure 2).^134^ A growing number of studies have shown that PI3K/Akt/mTOR pathway and Ras/Raf/MEK/ERK signalling pathway are associated with GF and GF receptors.^135^, ^136^ The PI3K/Akt signalling pathway also can inhibit GSK‐3β activity. Stimulation of PI3K can enhance NMDA‐dependent LTD, stimulate AMPAR exocytosis, and enhance excitatory synaptic transmission (Figure 2). More importantly, activation of the mechanistic target of rapamycin (mTOR) increases the synthesis of proteins required by existing synapse maturation and new synapse formation.^136^ Synaptic plasticity performs a central position in both short‐term and long‐term memory, and the mechanisms behind its changes are relevant to the pathophysiology and treatment of various psychiatric diseases.^136^

## 4 COGNITIVE DYSFUNCTION AND NEURON–GLIA INTERACTION

As the complexity, importance and diversity of glial cell functions are increasingly understood, there is growing interest in the potential for glial cell specialization and heterogeneity. Glial cells represent a range of non‐neuronal cells in the nervous system and comprise half of the volume of the human brain. A large body of evidence suggests that glial cells have an important influence on the development and structure of local neural networks, proving that higher brain functions are wrongly assumed to be exclusively neuronal activity. It is now believed that neuron–glia interactions are necessary for brain function.^137^ In the CNS, a large number of glial cells and neurons communicate with each other to perform elaborate brain structures and functions.^138^ Brain development, physiological function, and pathology are dependent on the close interaction between neurons and different types of glial cells. Glial cells (astrocytes, oligodendrocytes and microglia) and vascular cells (endothelial and pericytes) are as important to the development and function of the nervous system as neurons. Without glial cells, neurons cannot survive.^139^ Glial cells actively send signals to neurons and affect synaptic development, plasticity and transmission through a series of secretary and contact‐dependent signals. Intercellular connections in the nervous system are represented by chemical synapses, which are established mainly between neurons, and electrical synapses, which are established between glial cells.^140^ The pathophysiology of cognitive dysfunction is thought to be related to impairments in neural and synaptic plasticity, which in turn is modulated by the integrity of neuron–glial cells.^141^, ^142^ Alterations in glial cell number and/or function will influence the integrity and activity of the neuron–glial network, thereby affecting various behavioural outputs (e.g., cognitive function). Disruption of the glial signalling pathway can lead to synaptic and cognitive damage in disease.^141^ Glial cells restore vascularization, re‐establish the blood–brain barrier(BBB), stimulate synapse formation and complete remyelination mainly by promoting homeostasis of neural tissue after injury.^143^ Previous studies have shown that there is a cytokine network in the brain produced by neurons, microglia and astrocytes that deliver cytokine receptors and magnify cytokine signals.^144^ During development, microglia maintain a reservoir of oligodendrocyte progenitor cells (OPCs) and promote myelin formation. Microglia contribute to normal myelinogenesis and maintenance of oligodendrocyte progenitors in adulthood.^145^ Whereas activation of microglia was previously believed to be passive in response to neuronal damage, it is now thought that microglial activation may amplify ongoing neuronal injury and initiate additional neuronal loss. Neurons, glial cells, and immune cells from coordinated networks are involved in the pathogenesis of neuroinflammation and, more importantly, may also have a role in normal brain function. A complicated interaction network between neurons and the main three glial cells seems to be engaged in deciding the balance between pro‐and anti‐inflammatory signals, thus affecting the outcome of the immune response in the CNS. Glial cells not only provide support and nutrition to neurons but also protect the CNS from stress and pathogenic damage; however, excessive and prolonged glial activation result in more significant chronic neuronal damage, ultimately leading to neuroinflammation and neurodegeneration.^146^ Glial reactivity, glial atrophy and pathological remodelling are all essential elements of each psychiatric disorder, so further study of glial pathology may be critical to advancing new treatment approaches.^147^ Altogether, neuron–glia interactions have extensive implications for the targeted therapy of many different types of psychiatric disorders.

### 4.1 Neural and synaptic plasticity in cognitive dysfunction

Synaptic plasticity is the most fundamental function of the brain to perceive, evaluate and store complex information and to respond appropriately and adaptively to subsequent relevant stimuli.^148^, ^149^ Normal brain development is inextricably linked to the maintenance of neuronal plasticity and is highly dependent on the accurate response of neuronal cells to various stresses. Synapses are highly dynamic structures that can rapidly modify synaptic connections following neuronal activity and play an important role in improving cognitive performance. Synaptic deficits are the strongest correlative factor for cognitive decline in many neuropsychiatric diseases. The sustained increase and decrease in synaptic strength, known as LTP and long‐term inhibition (LTD), respectively, are generally considered to be associated with learning and memory and essential for normal cognitive function.^150^ The capability of weakening or strengthening synaptic connections between neurons, especially LTP and LTD, is one of the most dynamic areas of investigation in neuroscience.^151^ Studies have demonstrated that LTP is caused by the temporal and spatial coordination of multiple postsynaptic processes, including reorganization of the actin cytoskeleton, exocytosis of endosomes and insertion of AMPAR in synapses.^152^ NMDAR and AMPAR are key participants in excitatory neurotransmission and synaptic plasticity.^153^ Both LTP and LTD are involved in structural changes in dendritic spines. Dendritic spines are the primary site of information processing in the brain, and their morphological abnormalities are associated with cognitive dysfunction.^154^ Elevated Ca^2+^ concentrations in the spine trigger biochemical signalling that leads to the expression of multiple forms of synaptic plasticity. Considering the prominence of dendritic spines in synaptic transmission and plasticity, it is not surprising that disruption of dendritic spine plasticity leads to cognitive dysfunction in the brain. Glial cells are closely related to synapses and govern synapse formation, function, plasticity, and elimination of synapses during development and adulthood.^155^ There is accumulating evidence that glial cells influence major aspects of synaptic development, function and plasticity. Glial cells are critical elements that integrate and regulate neuronal activity and synaptic transmission. Disruption of glial signalling may lead to impaired cognitive function and result in pathological changes in synapses in the CNS. An increasing amount of research suggests that microglia and astrocytes may play a novel function in maintaining brain homeostasis, regulating synapse number, and neuroplasticity.^156^ Both extracellular microglia and astrocytes are well‐positioned to sense early disruptions in synaptic activity and cause synaptic death.^157^ Both extracellular microglia and astrocytes are well‐positioned to sense early disruption of synaptic activity and lead to synaptic death, and to mediate neuronal activity and synaptic plasticity. Microglia and astrocytes are important not only for normal brain development, but also for synaptic plasticity, and their applicability as a treatment for cognitive dysfunction has been widely discussed. Elucidating the possible mechanisms by which glial cells affect synaptic function may contribute to the development of new treatments for synaptic dysfunction.

### 4.2 Cognitive dysfunction and microglia

Glial cells are a variety of nervous system cells. In the mammalian brain, glial cells outnumber neurons and account for about half of the volume of the CNS. In the CNS, glial cells are usually divided into macroglia (mainly astrocytes and oligodendrocytes) and microglia.^158^ A century ago, microglia were identified as a distinct group of cells. It has long been thought that microglia are primarily mononuclear phagocytes from the mesoderm/mesenchyme responsible for removing debris during CNS development and disease.^159^ When the CNS or the brain is damaged or inflamed, the number of microglia increases and they then act like phagocytes to remove diseased cells. In addition to their phagocytic function, microglia also promote cells involved in synaptogenesis (synaptic formation and plasticity) in the death of developing neurons.^160^, ^161^, ^162^ With advances in imaging and genetics and the emergence of single‐cell techniques, microglia have been found to originate from uncommitted c‐Kit+ stem cells found in the yolk sac.^163^ Microglia make up 5%–20% of all glial cells in each region of the brain. Microglia are the primary line of defence against pathogens in the CNS and have the function of monitoring the brain parenchyma.^164^ Microglia are characterized by a rapid response to minor pathological changes in the CNS and are activated in almost all CNS diseases.^165^

Microglia are antigen‐presenting cells of CNS, which are part of the innate immune response, and can communicate with cytokine, chemokine, growth factor and other immune cells. Upon recognition of pathogens, activated microglia accumulate at sites of tissue injury and express inflammation‐related substances (e.g., proinflammatory cytokines, enzymes, adhesion molecules).^166^ Microglia contribute to chronic local inflammation and progressive neurodegeneration through the release of multiple inflammatory mediators.^167^ Both classical (M1) and alternate (M2) activation of microglia may be implicated in the pathogenesis of psychiatric disorders.^159^ When classical activation occurs, microglia exhibit the M1 phenotype, which is featured by pro‐inflammatory and pro‐killing functions. In the activated state of microglia, both secreted factors and cellular markers are dysregulated, and during M1 polarization, microglia release pro‐inflammatory cytokines. Previous studies have shown that macrophage colony‐stimulating factor and IL‐6 are key factors in microglia and astrocyte activation.^168^ The release of TNF‐α, IL‐1β by microglia activation has been demonstrated in various rodent models. In response to inflammatory stimuli, microglia produce large amounts of NO, which is generated by the activity of inducible iNOS. Under the condition of cognitive impairment, the activation of microglia induces the release of ROS and NO through activation of the NADPH oxidase, myeloperoxidase and iNOS. Altering intracellular ROS concentrations and NADPH oxidases have also been reported to activate microglia in response to additional stimuli. Oxidative stress and inflammation related to neuropsychiatric disorders may be the main contributors to NADPH oxidase and ROS production by microglia. In the inflammatory state, microglia rapidly upregulate the pattern recognition receptors (PRR) on the cell surface.

PRR expressed on the surface of microglia appears to be a common pathway for ROS production by multiple toxin signals. TLRs invoked microglia activation is responsible for the neurotoxicity of various CNS diseases. TLRs have been demonstrated to identify pathogen‐associated molecular patterns and trigger innate immune responses in their interactions with infectious agents.^169^ Studies have shown that LPS treatment leads to behavioural and cognitive impairments and neuroinflammation in mice that are associated with microglia activation and loss of hippocampal neuronal cells.^170^ Results indicate that as LPS causes proinflammatory in the hippocampus of prenatally stressed mice, mRNA levels of cytokines were significantly increased.^171^ Furthermore, prenatally stressed mice showed a greater ratio of hippocampal Iba1‐immunoreactive cells with morphological features of activated microglia than non‐stressed mice.^171^ CD11c + is a recently identified subpopulation of microglia, mainly found in the primary myelinated regions of the developing brain, expressing neuronal and glial cell survival, migration, and differentiation.^172^ Parkhurst et al. suggested that microglia exert an influential physiological role in learning and memory via the brain‐derived neurotrophic factor (BDNF) signalling pathway that promotes the formation of learning‐related synapses.^173^ As the function of microglia in detecting brain alterations becomes better understood, microglia activation in the progression and pathogenesis has been increasingly discussed. Insights into the mechanisms of brain–immune interactions can help us develop innovative strategies to activate microglia more efficiently whilst avoiding their deleterious effects on the components of neurons. There is no doubt that with the assistance of modern imaging and analytical techniques, microglia will illuminate the physiological functions of cognitive dysfunction in the years to come.

### 4.3 Cognitive dysfunction and astroglia

Astrocytes, derived from neuronal progenitors, are the most abundant and functional of the various glial cells.^116^ Astrocytes are also amongst the most specialized glial cells, outnumbering neurons by more than fivefold.^174^ Astrocytes are essential for regulating glucose metabolism, providing structural support, neurotransmitter uptake (especially glutamate), synaptic development and the BBB.^140^, ^175^ In the rodent models experiments, changes in the number and morphological phenotype of astrocytes have been shown to trigger cognitive dysfunction.^176^ Astrocytes are found throughout all regions of the CNS, mainly in grey matter and white matter. They are the most numerous and voluminous of the glial cells and are named after their stellate morphology.^167^ Based on their morphology and anatomical location, astrocytes can be divided into protoplasmic astrocytes and fibrous astrocytes.^174^, ^177^ Protoplasmic astrocytes are covered in all grey matter, whereas fibrous astrocytes are found throughout all white matter and take the form of many long fibrous projections. About 60% of the synapses in the CA1 region of the hippocampus are wrapped by protoplasmic astrocyte membranes.^178^

Astrocytes have the ability to regulate the structural remodelling and functional plasticity of synapses, so they are often regarded as the universal contributors to synaptic functions.^179^ Astrocytes actively control synaptic transmission by establishing synaptic connections with the brain during development to monitor and change synaptic function. Astrocytes can increase the number and postsynaptic activity of synapses by locating the AMPAR to the postsynaptic density.^179^ Astrocytes can modulate synaptogenesis by participating in synapse formation, regulating synaptic signalling and processing, releasing glial transmitters to regulate synaptic plasticity, providing metabolic and nutritional support to neurons and maintaining the balance of the internal environment of the nervous system. There is growing evidence that astrocytes have a regulatory role in synapse formation, transmission and plasticity. Stogsdill et al. have demonstrated that morphogenesis of astrocytes in the mouse cortex relies on direct interaction with neuronal processes and occurs simultaneously with the growth and activity of synaptic circuits.^180^ LTP and LTD are typical paradigms of the role of synaptic plasticity in astrocytes, which can rapidly regulate synaptic strength according to alterations in neuronal activity. Calcium signalling in astrocytes has been shown to affect the neuronal excitatory transmission, including network characteristics such as the ability to induce LTP. Recent work by Murphy‐Royal et al. has shown that stress impairs LTP by limiting shuttling of the astrocyte network, thereby impeding neuronal access to the astrocyte energy depots in the hippocampus and neocortex.^181^ Alterations in glial cell synapse or network function and abnormalities in astrocyte signalling may cause or contribute to synaptic and network imbalances, leading to cognitive impairment.^182^, ^183^

Astrocytes are directly involved in synaptic transmission by regulating intracellular calcium ion concentration and calcium signalling.^184^ Astrocytes express many neurotransmitter receptors and transporters, which may be activated by neurotransmitters released by synapses. Astrocytes have many neurotransmitter receptors that bind to intracellular calcium mobilization. Astrocytes can incorporate neuronal inputs, exhibit calcium excitability, and modulate neighbouring neurons via calcium‐dependent release of the chemical transmitter glutamate.^185^ Some evidence suggests that the Ca^2+^ to excitatory neuronal activity response of astrocytes is largely mediated by the activation of type I and type V metabolic glutamate receptors (mGluRs).^186^ Studies of experimental mouse hippocampal slices have shown that neuronal activity leads to increased calcium in glutamate‐dependent astrocytes. Recent studies suggest that delayed and deficient maturation of astrocytes may simultaneously lead to disruption of glutamate, potassium and neuroregulatory homeostasis, leading to impaired synaptic transmission.^187^ Understanding the action of astrocytes in sustaining glutamate homeostasis and modulating the homeostasis between glutamate uptake and release in the CNS contribute to our better understanding of the mechanisms of glutamate excitotoxicity in psychiatric disorders.^188^ Studies suggest that pathological processes targeting astrocytes may cause neurodegeneration in specific brain regions, which in turn may have an impact on cognitive function in mood disorders.^176^ The prefrontal cortex (PFC) exerts a key part in cognition, memory and emotional processing. Astrocyte loss (reduced glutamate uptake) in PFC is sufficient to induce anhedonia and depression‐like behaviour in rats.^189^, ^190^ Several drugs that enhance the functional activity of astrocytic glutamate transporters have been identified, such as β‐lactam antibiotic (ceftriaxone sodium), which show neuroprotective potential and positive results in several animal models of neurodegenerative and psychiatric disorders.^191^, ^192^

Changes in astrocytes are usually coordinated with alterations in spines, and dynamic structural changes in astrocytes contribute to controlling the extent of neuron–glia interaction at hippocampal synapses.^193^ Clinicopathological studies have shown that astrocyte density is decreased in the PFC of patients with major depression.^194^ Prevalent astrocyte markers (based on a variety of protein antibody positioning) in the brain tissue including GFAP, the water channel aquaporin‐4 (AQP4), the calcium‐binding protein, and the glutamatergic markers (including excitatory amino acid transporters 1 and 2 [EAAT1, EAAT2]) and glutamine synthetase, etc.^175^, ^195^ There is evidence that each of these astrocyte markers is altered in psychiatric disorders. In addition, the lamina‐specific GFAP response in the dlPFC astrocytes is reduced in patients with schizophrenia.^196^ Reduced astrocyte numbers, morphological atrophy, decreased GFAP expression, as well as inadequate glutamate uptake, are manifestations of cognitive dysfunction in a variety of psychiatric disorders.^197^, ^198^, ^199^ Astrocytes are intensively implicated in several aspects of the CNS function, including oxidative stress regulation. In certain pathological conditions, astrocytes may be one of the main sources of detrimental ROS and RNS. Increasing evidence suggests that astrocytes may be a prospective target for the modulation of oxidative stress in the CNS, which may provide us with a future approach for the treatment of related diseases. Numerous studies have shown that mitochondrial energy damage is a key determinant of astrocyte sensitivity to neurotoxic processes. Calabrese et al. suggested that LPS/INF‐γtreatment‐induced hsp70 (heat shock protein 70) stress protein expression in astrocytes, which provided evidence that iNOS induction in astrocyte culture was related to the activation of the stress pathway.^200^ In addition, due to the different roles of astrocytes in various conditions, individualized interventions for patients need to be considered. Understanding the emerging role of astrocytes in cognitive dysfunction will provide us with some new therapeutic opportunities.

### 4.4 Cognitive dysfunction and oligodendrocytes (OLs)

In terms of numbers, the most numerous glial cells are oligodendrocytes and NG2 cells (40%–60%).^201^ Oligodendrocytes are the core of myelinated Schwann cells.^185^ Oligodendrocytes are best known for their role in myelination. Myelination is a dynamic process that responds to external stimuli, which is consistent with the activity of neural networks that mature long after ontogeny.^187^ Several experiments in rodents have shown that oligodendrocytes are particularly sensitive to alternations in the neural environment during critical windows of individual development when developing myelin formation is most likely to be disrupted.^187^ Steadman's study showed that disruptive oligodendrogenesis reduces myelin formation and impairs spatial memory consolidation.^202^ Oligodendrocytes and myelin are vital for rapid neuronal signalling and also supply metabolic and nutritional support for axons, and their damage causes axonal death and neurodegeneration, which are critical characteristics of AD.^203^ Oligodendrocytes maintain normal axon function by myelinating the axons, and oligodendrocyte injury leads to Waller degeneration, which inevitably leads to axonal demise.^139^ Myelinated axons not only transmit information more rapidly than unmyelinated axons but also transmit information more coherently. The oligodendrocytes wrap axons with myelin sheaths to support the local metabolic structure and homeostasis of axons.^204^ Oligodendrocytes are a dominant cell type of white matter (approximately 50% of the total volume of the human brain) and express glutamate receptors and transporters.^139^, ^205^ Altered expression of NMDA receptors (NMDARs) and enzymes involved in glutamate metabolism has been found in schizophrenic patients.^206^ Neuronal activity is a key determinant of myelination, therefore, myelination can be reduced by inhibiting neuronal NMDARs and inhibiting action potential.^207^

NG‐2 glial cells, also named oligodendrocyte precursor cells (OPCs) or polymorphic glial cells, are homeostatic and facilitate myelination in adulthood, although their function has not been better described.^208^OPCs originated in a specific region of the developing CNS, and once they are generated, they migrate, differentiate and mature in a specific region of oligodendrocytes. OPCs retain the ability to proliferate, migrate and differentiate into oligodendrocytes.^209^ OPCs in the adult CNS form synapses with neurons, support the integrity of the BBB and mediate neuroinflammation. In mice with subcortical ischemic vascular dementia (SIVD), a model of cognitive dysfunction, Ohtomo et al. found that cognitive function decreased significantly, but improved after 6 weeks of treadmill exercise, with an increased number of OPCs in the white matter.^210^ Experimental studies by Wang et al. showed that myelination was very active in young mice, whereas spatial memory was greatly inhibited in elderly mice, indicating that spatial learning and memory require myelination and that promoting myelination can improve ageing‐induced decline in memory function.^211^ This suggests that promoting myelination may be a therapeutic strategy to alleviate cognitive function. Several studies have shown breakthroughs in the involvement of oligodendrocytes in learning, memory and cognition, but there is still a long way to go, and many exciting cellular mysteries remain to unravel.^212^

## 5 COGNITIVE DYSFUNCTION AND STRESS

Stress is a significant contributor to the risk of psychiatric disorders, and the progression and aggravation of depression and anxiety disorders are associated with recurrent psychosocial stress.^213^ Psychological stress is a very prevalent life event that often affects brain function and cognitive performance, and the immune system has a supportive role in brain plasticity and homeostasis. The brain is a crucial organ for interpreting and coping with potential stress and serves an essential role in controlling the generation of stress and its behavioural and physiological reactions. Changes in the brain and immune system induced by acute and chronic stress and their potential mechanisms have been shown to possess unintended clinical results. Studies have shown that stress affects cognitive function in multiple ways and that the effects of stress on cognition involve multiple mechanisms and different time courses.^214^

Preclinical studies have shown that stress paradigms, such as chronic unpredictable stress, social defeat and learned helplessness induce pro‐inflammatory cytokines in the central and peripheral areas, and that these changes are associated with depression‐like symptoms in psychiatric disorders.^215^, ^216^ Activation of the inflammasome and elevated cytokines during stress and depression suggest that inhibition of inflammatory response can alleviate depressive symptoms.^217^ In an experimental study, it was found that adult male rats exposed to prenatal stress showed increased expression of cytokines in the hippocampus, and these cytokines were more likely to activate microglial and astrocyte responses in adulthood.^218^ A person's ability to cope with stress is primarily regulated by the HPA axis, which is self‐regulating under normal physiological conditions and its activity is reduced by negative feedback inhibition. Elevated stress hormones directly affect the motility of various types of cells in the immune system.^219^ In particular, stress hormones and glucocorticoids may lead to damaged cognitive function and contribute to impairment of brain structures.^220^ In general, when physiological stress causes neuroinflammation, glucocorticoids exert anti‐inflammatory effects by increasing levels of anti‐inflammatory cytokines and decreasing levels of pro‐inflammatory cytokines.^221^ Stress can reduce the number of axon‐spinous excitatory synapses and may change the synaptic morphology.^222^ The stress response stimulates specific neural circuits in the hippocampus, the PFC, and the amygdala. Even very mild and acute stress can cause rapid cognitive impairment in the PFC (the region most sensitive to the harmful effects of stress exposure), whilst prolonged stress exposure could lead to structural changes in the dendrites of the PFC.^223^ Treatment with fluoxetine prevented stress‐induced reductions in astrocyte numbers, whereas in non‐stressed animals, fluoxetine did not affect astrocyte numbers.

### 5.1 Cognitive dysfunction and chronic stress

Since the early 1990s, several neurobiological studies have emphasized the effects of chronic stress on the developing brain.^224^ Unavoidable chronic stress will eventually lead to changes in mental status and pathological changes in the function of the immune system, ultimately causing irreversible damage to the body.^225^ The risk of chronic exposure to stress hormones, whether occurring in prenatal, infancy, childhood, adolescence adulthood or old age, affects brain–immune interactions involving cognitive and mental health.^226^ Chronic unpredictable stress (CUS) has been shown to induce changes in amino acid neurotransmitter metabolism in glial cells.^189^ Studies have shown that chronic inflammation is caused by stress and disturbances in the activity of pro‐inflammatory cytokines. Under severe or chronic stress situations, the immune system is intensely stimulated, and glial and other immune cells in the brain alter their morphology and function and secrete high levels of pro‐inflammatory cytokines. CUS is a rodent model of depression, and a series of experimental evidence by Banasr et al. shows that chronic stress can damage the glial function occurring in the rat PFC.^189^ Immunohistochemical results show that chronic stress dramatically reduced the overall number of GFAP‐positive astrocytes in the mouse/rat hippocampus. There is considerable evidence that long‐term stress exposure results in permanent loss of neurons in the hippocampal region, as well as long‐term changes in dendritic structure.^214^ There is also evidence that chronic stress‐induced glial cell dysfunction leads to elevated extracellular glutamate levels with neurotoxic effects, resulting in astrocyte density and a decrease in GABAergic interneurons.^195^, ^227^ Interventions such as regular physical activity, regular diet as well as positive social support as an adjunct to medication, can reduce the long‐term burden of stress and benefit brain and body health and resilience.

### 5.2 Cognitive dysfunction and post‐traumatic stress

Traumatic stress leads to a variety of psychiatric disorders, including depression, anxiety, and trauma‐related disorders, especially post‐traumatic stress disorder (PTSD). PTSD is highly correlated with immune dysregulation, and several studies have demonstrated that stress exposure enhances fear learning (SEFL), which is related to increased hippocampal IL‐1β in preclinical animal models.^227^ Research by Risa Imai et al. would suggest that serum inflammatory cytokine IL‐6 levels in female individuals with PTSD are dramatically higher than those in healthy controls,^228^ which supports the hypothesis of neuroinflammation in cognitive dysfunction. The results of meta‐analyses showed that IFN‐γ, TNF‐α, and the C‐reactive protein were elevated in PTSD.^229^ Postoperative cognitive dysfunction (POCD) is a neurological disease associated with neuroinflammation and is the most common type of cognitive dysfunction and has been widely reported after various surgical operations and is particularly common in elderly patients.^230^, ^231^, ^232^ Postoperative systemic and hippocampal inflammation is a key factor in the pathogenesis of POCD.^233^, ^234^, ^235^, ^236^, ^237^ It has been shown that noticeably increased expression of GFAP, that is, activation of astrocytes, in the hippocampus of mice with the POCD animal model group can cause cognitive dysfunction. There are three main strategies for the treatment of POCD: blocking inflammation (anti‐inflammatory) by inhibiting inflammatory mediators, preventing oxidative components of inflammation from oxidizing (antioxidant), and protecting neurons during surgery.

### 5.3 Cognitive dysfunction and early‐life stress (potential neuro‐immunological mechanisms)

The sensitive window of time early in life is a critical period for brain development. Both animal and human studies have found that stressful life events, exposure to natural disasters, and symptoms of maternal anxiety and depression can affect the brain and increase the risk of offspring facing a range of emotional, behavioural and cognitive problems later in life.^238^ During this sensitive time window in early life, stress may influence patterns of emotional and stress responses throughout life, altering the rate of brain and body ageing and ultimately leading to psychiatric disorders and neurodegenerative diseases. Research suggests that even minor activation of the immune system early in life may increase susceptibility to a range of psychiatric disorders and lead to physiological abnormalities in the body.^239^ Early‐life stress (ELS) significantly increases the risk of developing psychiatric disorders such as MDD, schizophrenia, and PTSD, all of which are characterized by cognitive dysfunction.^240^ Early adversity is linked to deficits in a wide variety of cognitive and emotional functions.^241^ Namely, early life experiences, such as maternal deprivation, exposure to infections, and medicine use, can lead to permanent alterations of neural and immune system function and can affect brain structure and function, which are critical for cognitive function, emotional behaviour, and neuroplasticity in adolescence and adulthood.^242^ Epidemiological data indicate that ELS occurs in different age groups, with the youngest age group of 1–3 years being the most affected.^243^ Studies have shown that nearly 32% of psychiatric disorders are related to adverse childhood experiences.^244^ Experiments in rodents have shown that ELS increases stress sensitivity in adult mice during the postnatal sensitive period of female mice.^245^ ELS is a fragile factor in the progression of psychiatric disorders, modulating inflammatory responses and influences neuroplasticity mechanisms throughout the life span.

High levels of pro‐inflammatory cytokines produced by the maternal or foetal immune system during perinatal infection are related to an elevated risk of foetal brain abnormalities as well as neurodevelopmental disorders. Inflammation appears to play an important role in many ELS, and several research groups have suggested that elevated levels of pro‐inflammatory cytokines mediate the long‐term consequences of many ELS.^246^, ^247^ Studies by Diz‐Chaves et al. showed that prenatal stress influences the inflammatory response in the hippocampus of adult male mice.^218^ Prenatally stressed mice show higher levels of IL‐1β and TNF‐α in the hippocampus and an elevated proportion of microglia with reactive morphology in hippocampal CA1.^218^ Besides, prenatally stressed mice treated with LPS exhibited elevated TNF‐α immunoreactivity in CA1, as well as elevated numbers of the ionized calcium‐binding adaptor protein‐1(Iba‐1) immunoreactive microglia as well as GFAP immunoreactive astrocytes in the dentate gyrus.^218^ ELS has been shown to interfere with microglia developmental processes, including their proliferation, death and phagocytic activity. Elevated corticosterone levels in the ELS mouse model (mice briefly exposed to a daily absence in the first weeks after birth) interfere with microglia function during a critical time in brain development.^247^ The brief daily separation (BDS) (exposure in the first few weeks of life) is the most commonly used mouse model of ELS, which results in elevated corticosterone levels that impair microglial function during critical periods of brain development. Recent studies have shown that BDS interferes with the function of developing hippocampal microglia whilst impairing several microglia‐mediated developmental processes (e.g. synaptogenesis, synaptic pruning, the growth of axons, as well as myelination).^248^ Maternal sleep deprivation (MSD) that deprived pregnant Wistar rats of 72 h of sleep in the last trimester of gestation, an increase in Iba1(+) microglia, increased in number and decreased in several hippocampal neurons.^249^ Recent work by Cao et al. has shown that inflammation early in life leads to dysregulation of microglial phagocytosis in the anterior cingulate cortex (ACC), which in turn leads to the excessive phagocytic activity of neuronal dendritic spines, as well as diminished resistance of ACC glutamatergic neurons to stress, leading to various depressions in adolescence.^250^

ELS primes glial cells, leading to an enhanced response to subsequent stress.^251^, ^252^, ^253^, ^254^ Regulation of microglia is associated with ELS/prenatal stress as well as stressors in adulthood. It has been shown that ELS disrupts a variety of developmental processes regulated by microglia, mediates some developmental abnormalities in BDS mice and alters the activity of crucial transcription factors in postnatal microglia.^248^ During ELS, the neurodevelopment and synaptic plasticity in the brain are altered, both of which are extremely important in regulating cognitive function. The work of Makinodan et al. suggests that the effects of childhood neglect and isolation on adult mental health may be at least partly caused by changes in oligodendrocytes and myelin sheath development.^255^ Because of the marked plasticity of the brain and immune system, many of the detrimental effects of ELS can be alleviated by considering interventions that target neuroimmune interactions over the lifespan.^256^ ELS‐induced brain and immune system changes and their underlying mechanisms have been shown to have unintended clinical consequences. ELS increases the response of microglia and astrocytes to pro‐inflammatory cytokines, leaving brain structures in an inflammatory state.

## 6 COGNITIVE DYSFUNCTION AND PSYCHIATRIC DISORDERS (FOCUS ON TREATMENT)

Traditionally, research on psychiatric disorders has mainly concentrated on psychological symptoms, such as depression, pessimism, anxiety and delusions.^257^ An increasing number of researchers are now listing cognitive dysfunction as the primary cause of a range of psychiatric disorders in addition to emotional symptoms,^1^, ^2^, ^258^ it underscoring the necessity to treat cognition dysfunction separately. There is considerable evidence that persistent cognitive impairment may affect the resilience of patients with psychiatric disorders,^259^ and it is generally accepted that individuals with cognitive dysfunction tend to suffer from long‐lasting distress.^1^ Cognitive dysfunction is a major symptom of various psychiatric disorders such as depression, schizophrenia and PTSD, as well as a series of neurodegenerative disorders such as AD and PD (Figure 3). The classic inflammatory cytokines IL‐1β and TNF‐α affect the pathophysiological processes of MDD, AD and PTSD. Chronic microglial activation and associated neuroinflammation are frequently observed in animal models of neurodegenerative diseases. Postmortem analysis of tissues from psychiatric patients has shown a distinct decline in the number of glial cells in brain regions closely associated with psychiatric disorders. Despite the aetiology and pathogenesis of cognitive dysfunction remain incompletely elucidated, the pathological processes of brain–immune interaction appear to contribute to its treatment (Table 1). Therefore, studying brain–immune interactions may help us to understand the pathogenesis of cognitive disorders and ultimately to develop more effective treatment strategies for various psychiatric disorders.

**TABLE 1:** Symptoms, aetiology, the average age of onset immune response, and possible therapeutic methods of common psychiatric disorders

Disease	Core symptoms	Additional symptom	Possible aetiology	The average age of onset	Incidence	Innate immune response involvement	Adaptive immune response involvement	Therapeutic intervention
PTSD	Increased alertness; avoidance behaviour; numb; complex hallucination/delusions	Cognitive dysfunction; depression; sleep disorders	Multifactorial (neuroimmune, neuroinflammation)	Variable	Occurs in 5%–10% of the population^260^	↑Pro‐inflammatory cytokines, ↑chemokines, microglial activation^261^	Not reported	Mindfulness‐based cognitive therapy; cytokine /chemokine modulation^262^
MDD	Low mood; despair; sleep disturbance; inattention; hypaphrodisia; anhedonia; lacking strength; apathy	Cognitive dysfunction	Multifactorial (neuroimmune, neurogenesis, genetics)	All period of life	About 2.4%–3.8% lifetime prevalence^263^	↑Cytokines, ↑chemokines, microglial activation	↑T‐reg cells	Innate immunity based therapy (e.g. inhibiting/modulating microglia phenotypes, inhibiting NLRP inflammasome), antioxidants
SCZ	Impaired motivation, delusions; depressive/elated mood; social withdrawal; learning and attention disorders	Cognitive dysfunction	Multifactorial (e.g. dopaminergic hypothesis, neuroimmune, neuroinflammation)	15–25 years in males and 16–30 years menopausal age in females^264^	Affects up to 1% of the population^265^	↑Pro‐inflammatory cytokines, ↑chemokines, ↑TLRs, ↓NK cells, ↑ROS, microglial activation	Not reported	Cytokine /chemokine modulation, antioxidants
AD	Cognitive dysfunction, the decline of intellectual function, memory loss, executive function decline, inattention, anxiety, apathy	Cognitive dysfunction; epilepsy	Multifactorial (e.g. accumulation of amyloid‐beta plaques, hyperphosphorylated tau in neurofibrillary tangles, neuroinflammation)	60–85 years (younger trend)^266^	Quadruple by the year 2050^267^	↑TNF‐α, ↑IFN‐γ, ↑chemokines, ↑complement, ↑TLRs, activated/ dystrophic microglia	↑T‐cell response and antibody	Stem cell therapy, antibodies to abnormal protein aggregations, non‐steroidal anti‐inflammatory, complement inhibition, reducing NLRP‐3 Inflammasome
PD	Tremor, gait rigidity, hypokinesia, depression, anxiety, anhedonia, sleep disorders, neurobehavioural abnormalities	Cognitive dysfunction	Multifactorial (e.g. selective loss of dopaminergic neurons in the substantia nigra pars compacta due to α‐syn‐intraneuronal inclusions)^268^	The average age of onset was about 60 years	Double in 25 years^269^	↑IL‐1β, ↑IL‐6, ↑TNF‐α, ↑TLRs, activated NK cells, microglial activation, structural changes in astrocytes	↑T‐cells	Stem cell therapy, Mediterranean diet or a plant‐based diet, regular exercise^270^

### 6.1 Cognitive dysfunction and MDD

Cognitive dysfunction is increasingly recognized as a core feature of MDD, with deficits observed in several domains (e.g., learning, memory, attention, executive function).^259^ Although the specific neurophysiological characteristics of cognitive dysfunction in MDD have yet not been fully elucidated, a host of research suggests that patients with MDD have neurocognitive deficits in the cognitive domains.^258^ There is ample evidence that cognitive dysfunction may persist following the remission of a major depressive episode.^10^, ^175^ A growing number of researchers believe that depression and inflammation are intertwined, with depression leading to inflammation, and inflammation‐promoting depression, and that this two‐way bidirectional cycle has a major impact on maintaining the health of patients with psychiatric disorders.^271^ There is a growing body of compelling evidence that communication between the peripheral and the brain immune systems may lead to brain inflammation, which in turn leads to impaired neurogenesis and neuroplasticity damage, ultimately leading to MDD. Preliminary data from patients with autoinflammatory disorders and depression suggest that inhibition of pro‐inflammatory cytokines or their signalling circuits leads to improved depressed mood and increased response to traditional antidepressant treatment.^18^ Many meta‐analyses have reported higher concentrations of pro‐inflammatory cytokines in patients with depression than in controls.^272^, ^273^, ^274^ Rising evidence of preclinical and post‐mortem studies shows decreased glial cell number or density and morphological and functional glial atrophy in MDD patients and animal models of depression.^275^, ^276^, ^277^, ^278^ The current preclinical studies suggest that astrocytes and glial fibrillary acidic protein (GFAP) are involved in animal models of stress and depression‐like behaviour.^175^, ^271^ The diverse astrocyte deficiencies noted in MDD suggest that astrocytes may be a new target for the antidepressant effect. Researchers have suggested astrocytes as a target for therapeutic intervention in depression, and several animal studies have shown that different classes of antidepressants affect astrocytes, such as fluoxetine, which prevents psychological stress‐induced astrocytes reduction.^279^ Thus, there is an increasing interest in treating depression from the perspective of brain–immune interaction mechanisms.

### 6.2 Cognitive dysfunction and schizophrenia (SCZ)

SCZ is a complex, heterogeneous behavioural and cognitive psychiatric disorder with distinct damage to psychosocial functioning.^280^, ^281^ Although cognitive dysfunction is considered a central feature of SCZ, little is known about its underlying pathophysiology. SCZ may be linked to an imbalance of inflammatory cytokine, resulting in a decrease in Th1 and an increase in cytokine secretion by Th2.^282^ Mounting evidence shows that inflammation leads to the pathogenesis and progression of SCZ, and therapeutic agents with anti‐inflammatory or neurotrophic effects may be beneficial in the treatment of SCZ.^283^ Numerous studies have shown that plasma amounts of pro‐inflammatory factors are considerably elevated in patients with schizophrenia compared to controls.^283^, ^284^ Meta‐analysis of anti‐inflammatory treatment of mood disorders has shown that anti‐inflammatory treatment reduces the number of patients receiving post‐treatment manic symptom scores and post‐treatment depressive symptom scores in patients receiving anti‐inflammatory drugs.^285^, ^286^ Studies also suggest that learning and memory impairment in schizophrenia may be caused by pro‐inflammatory cytokine activity produced by microglia,^287^, ^288^, ^289^, ^290^, ^291^ and the microglia inhibitor minocycline may block the cytokine damage to the hippocampus and reverse the cognitive dysfunction in the SCZ.^292^ The microglia hypothesis of SCZ may provide new ideas for treatment strategies for SCZ.^290^ Recent research by Yilmaz et al. has shown that significant loss of grey matter and dendritic spines occur during the onset of SCZ, suggesting that decreased synaptic connections may contribute to behavioural and cognitive dysfunction.^293^ However, it remains unclear when the loss of synapses in the cerebral cortex occurs and how synaptic the relationship is between the loss of connections and the proteins they encode. It has been suggested that the SCZ may be caused by dysregulation of the normal function of astrocytes on synapses. Furthermore, the dysregulation of processes associated with oligodendrocytes in SCZ provides substantial evidence for irregular expression of myelin dysregulated genes and altered oligodendrocyte numbers in the brains of SCZ patients.^294^ Defects in oligodendrocytes in SCZ patients may be the result of maturation failure and disturbed regeneration, which may underlie cognitive deficits in the disease and is closely associated with impaired long‐term outcomes.^294^ Therefore, treating SCZ from the perspective of synaptic plasticity and neuron–glia interaction, namely the mechanism of brain–immune interaction, may be one of the future directions.

### 6.3 Cognitive dysfunction and AD

AD is one of the common senile diseases that can lead to a decline in intellectual function with cognitive impairment and memory loss, which can seriously affect the quality of life of patients. However, there are few treatment strategies for AD and it is difficult to cure, so it is critical to understand the pathogenesis of AD. Accumulation of amyloid‐beta plaques, hyperphosphorylation of tau proteins in neurofibrillary tangles, and neuroinflammation are possible mechanisms of AD.^295^ There is growing evidence that the pathogenesis of AD is not limited to the prevailing amyloid cascade hypothesis and microtubule‐associated protein (tau‐a microtubule‐associated protein) dysfunction hypothesis, but interacts with immunological mechanisms in the brain.^296^ Neuroinflammation is one of the fundamental features of AD, but its exact role in disease progression remains unclear. A growing body of brain imaging data is available to support the contribution of neuroinflammation in AD progression.^297^ Several studies have also shown elevated levels of cytokines and chemokines in the brains of AD individuals and proliferation of microglia in damaged regions.^298^ Several studies have also shown higher levels of inflammatory cytokines in AD patients compared to controls.^299^, ^300^ In addition, recent studies suggest that novel anti‐inflammatory agents for instance minocycline (a tetracycline derivative) may exert neuroprotective action in the treatment of AD.^301^ More and more studies also suggest that glial cells are one of the main drivers of neuroinflammatory processes,^302^ and regulating glial cells could be a strategy for the treatment of AD.^303^ In the AD, a cluster of reactive microglia surrounds senile plaques, and there is growing evidence that microglia proliferation and activation in the brain is a prominent feature of AD, and that impaired microglia activity and altered microglia response to β‐amyloid (Aβ) are correlated with an increased risk of AD.^304^ Aβ proteins are also widely used to activate microglial cells in vitro. Krasemann et al. demonstrated that the TREM2‐APOE circuit is a principal modulator of the functional phenotypes of microglia in psychiatric diseases and a novel objective to assist in restoring microglia homeostasis.^305^ A family of nucleoside‐binding oligomeric domain‐like receptors, pyrin domain‐containing‐3 (NLRP3) inflammasome, is one of the features of the neurodegenerative disease AD.^306^ Recent studies suggest that NLRP3 may be used for targeted treatment to reduce neuroinflammation and that modulating endogenous cellular defence mechanisms might be an innovative method for the treatment of interventional AD.^307^ Studies also suggest that microglia, as the major participants in neuroinflammation, may be modulated differently in AD locus to prevent or improve disease progression.^296^ Enhancing autophagy in microglia may be a promising new therapeutic strategy for AD.^308^ Astrocytes are active participants in AD. The accumulated astrocytes around plaques and neurofibrillary tangles are a hallmark of AD. Astrocytes produce a wide diversity of chemokines to recruit microglia and pro‐inflammatory cytokines to promote neurodegeneration. Astrocytes can secrete anti‐inflammatory cytokines, soluble factors, and BDNF, which can inhibit the activation of T cells and microglia. As previously mentioned, reactive astrocytes produce a variety of molecules with pro‐inflammatory or anti‐inflammatory potential under different stimuli, and increased expression of GFAP and hypertrophy of astrocytes near amyloid plaques are some of the well‐known characteristics of AD.^309^, ^310^ By releasing pro‐inflammatory cytokines, astrocytes cause the recruitment of autoimmune cells and the destruction of oligodendrocytes, leading to the onset of psychiatric disorders. Changes in the number and/or dysfunction of oligodendrocytes or their precursors can affect the integrity of myelin, and repair of myelin injury may be an important early protocol for AD.^311^ Astrocyte and oligodendrocytes are particularly promising in cell replacement therapy. With the development of stem cell technology and the ability of stem cells to generate various types of neurons and glial cells, stem cell therapy promises to be a new approach for the treatment of AD.^312^ Recent breakthroughs in clinical trials with stem cells hold promise for curing refractory psychiatric disorders.^312^ Neural stem cell (NSC)‐based treatments for AD have been demonstrated in several animal models.^313^, ^314^

### 6.4 Cognitive dysfunction and PD

PD is considered one of the most prevalent age‐related neurodegenerative disorders of the CNS.^315^ PD is closely related to neurobehavioural disorders (anxiety, depression), cognitive dysfunction (dementia), and autonomic nervous dysfunction (such as hyperhidrosis).^316^ Several longitudinal studies have indicated that mild cognitive dysfunction is a harbinger of PD.^315^ Recent investigations have revealed that higher levels of inflammatory biomarkers correlate with cognitive impairment in patients with PD.^317^ Some data suggest that PD is connected with a proinflammatory profile, and soluble TNF receptors (sTNFR) are recognized biomarkers of cognitive performance.^318^ Molecules that can activate the anti‐inflammatory M2 phenotype or facilitate the conversion of the pro‐inflammatory M1 phenotype to the anti‐inflammatory M2 phenotype can be used to treat PD.^319^ Anti‐inflammatory molecules exert neuroprotective effects by modifying the balance of M1 and M2. In a mouse PD model of MPTP (a byproduct of methotrexate analogue, a neurotoxin), infusion of human AVV expressing IL‐10 reduced the expression of pro‐inflammatory iNOS and significantly increased the levels of anti‐inflammatory mediators.^319^, ^320^ MPTP triggers an inflammatory response that leads to neurodegeneration, causing microglia activation and M1‐related increases in pro‐inflammatory cytokines.^319^ There is growing evidence that PD is associated with elevated TNF‐α levels, which are markedly elevated in the cerebrospinal fluid and postmortem brain in PD sufferers. Inflammation is thought to be an early‐stage event in PD, so it may be more productive to target inflammatory pathways to prevent disease progression. Therefore, immunoregulatory therapies are currently recognized as a neuroprotective strategy for PD.^321^ The pathogenesis of PD is characterized by the activation of microglia. Increased microglial activation is considered to lead to cell death in PD, and inflammation markers may be biomarkers for its prognosis.^322^ Recent studies have shown that the NLRP3 inhibitor MCC950 effectively inhibits the activation of the microglial inflammasome and reduces motor defects in a variety of PD models.^321^ A healthy lifestyle (such as the Mediterranean diet or a plant‐based diet and regular exercise) may reduce chronic inflammation and positively affect PD symptoms and even disease progression, and this strategy may be an effective, non‐pharmacological treatment strategy.^270^

## 7 CONCLUSION

Neuropsychiatric disorders involve complex cellular and molecular processes, and cognitive dysfunction is commonly considered to be a pivotal part of many psychiatric disorders. With an understanding of the molecular and cellular mechanisms underlying cognitive dysfunction has increased, many researchers believe that cognitive dysfunction depends on the bidirectional regulation of the neural and immune systems. Research on the cognitive decline has focused on the regulation of several crucial neuro‐immune physiological processes, including immune molecular, neuroglial, oxidative stress as well as early‐life stress. An increase in ROS (oxidative stress response) is associated with loss of mitochondrial function and reduced antioxidant defences in psychiatric disorders, which can directly affect synaptic activity and neurotransmission, causing cognitive dysfunction. Excessive formation of ROS/RNS leads to oxidative stress and damages cells and tissues and regulates intracellular signalling cascades that contribute to the development of psychiatric disorders. Altered redox homeostasis accompanied by ROS/RNS is closely associated with cognitive dysfunction in psychiatric disorders. Activation of the brain axis by vitagene, cellular protection preconditioning signal, is an important redox‐dependent regulator affected by free radical oxygen and nitrogen species. The redox‐active enzymes involved in the neurogasobiology of central and peripheral CNS stress tolerance are relevant to neurodegenerative/ neuroprotective mechanisms. The complex role of ROS/RNS‐induced redox signalling in inflammation will contribute to the interpretation of therapeutic intervention strategies related to psychiatric disorders. Inflammatory factors may regulate synaptic plasticity and induce neurotransmission through the crosstalk between the nervous and the immune systems. Microglia and astrocytes play a role in cognitive processes and may regulate neurogenesis at the cellular level. In addition, alterations in early‐life stress are associated with a wide range of cognitive‐related disorders. The regulation of these important factors depends on the comprehension of the mechanisms of brain–immune interaction. A growing body of evidence suggests that brain–immune interaction mechanisms perform a pivotal role in cognitive dysfunction. This article reviews how various factors involved in the brain–immune interaction mechanisms modulate the cognitive function and ultimately lead to cognitive dysfunction in the brain. Although multiple factors may partially explain the brain–immune interaction mechanisms underlying cognitive dysfunction. However, the specific mechanism of brain–immune interaction in cognitive dysfunction is unclear and needs to be further investigated. In conclusion, we aimed to briefly summarize the role of brain–immune interaction mechanisms behind cognitive dysfunction to provide effective treatment strategies for various psychiatric disorders.

## AUTHOR CONTRIBUTIONS

Fangyi Zhao wrote the manuscript. Wei Yang and Tongtong Ge provide the construction. Bingjin Li and Ranji Cui provide the final revision.

## CONFLICT OF INTEREST

The authors declare no conflict of interest.

## Data Availability

No data, models, or code were generated or used during the study.
